# Alloys of Commerce and Their Metal Formulæ

**Published:** 1904-05-15

**Authors:** N. K. Garhart


### ALLOYS OF COMMERCE AND THEIR METAL FORMULAE.

BY N. K. GARHART, PH. G.

Read before the Central and Southwestern Michigan Dental Societies, April 12, 1904.

Before taking up the subject matter of my paper, let us review some of the important discoveries which careful experimental work has so far revealed to us. We find from the results of Dr. Flagg's labors the urgent need of more silver in our alloys. When Dr. Black appeared on the scene with a more scientific method of research, the results of his work profoundly impressed the dental profession. He also found that the average dental alloy could be vastly improved in edge-strength by incorporating higher percentages of silver into its formula. Modern engineering terms, such as "flow" and "stress," were adopted to emphasize the importance of increasing the edge-strength of our alloys. He contended that alloys of the "old school" or forty to fifty percent silver formulæ, were not of sufficient hardness to withstand the stress of mastication. So far as this portion of his work is concerned, he greatly overestimated the ordinary force of mastication. However, he did not overlook the supreme issue of the day, namely, shrinkage. Alloys always did shrink up to the time of the first appearance of the quick-setting formulæ. The first step taken to eliminate this defect was accomplished by increasing the percentage of silver. Dr. Black found that something more than the mere increase in the proportion of silver was necessary to overcome the shrinkage of alloys. His experimental work revealed the fact that small percentages of copper and zinc judiciously used in conjunction with the proper proportions of silver and tin would produce a slightly expanding amalgam. He further observed that filed alloys gave better results than if the same formulæ was cut in the form of shavings. He never did give a satisfactory explanation why this difference should exist. In order that I might obtain some more information on this subject, I prepared six different alloys, each one of which were cut in four different thicknesses of shavings. Cut No. 1 was the thinnest, while the other three were progressively twenty-five percent thicker. Measurements of the shrinkage and expansion present in the various alloys were carefully recorded. My records revealed a marked decrease in the volume of shrinkage present for every increase in the thickness of shavings. It will be observed that all alloys should be cut on automatic cutting machines, the proper thickness of cutting having first been determined by careful experimentation for each alloy. We find so many alloys of commerce that are unevenly cut, others cut too coarse, while a good many others are shaved too thin.

Alloys of commerce and their metal formulæ should be a very interesting subject, considering that so many manufacturers are of the belief that their interest would be jeopardized should the exact formulæ of their products be revealed to the profession. They claim that their secret processes of manufacture make it impossible for chemists to disclose the exact formulæ of their products. There are other claims equally ridiculous, such as the use of a certain kinds of bronze or alloy which makes the amalgam keep its color as gold and causing it to adhere to the walls of the cavity.

It would take up too much valuable time for me to recite the various analytical methods that were employed in this work. I have this detailed information at hand and it may be had for the asking. Each assay was carried out in duplicate form in order that no error might creep into my work. Each alloy was tested for the presence of the metals, tin, silver, copper, zinc, gold and platinum, with the following results:

| | Silver. | Tin. | Gold. | Copper. | Zinc. |
|---|---|---|---|---|---|
| Globe, (S. S. W.) | 44.89 | 51.90 | 2.71 | ----- | .50 |
| Splendid, (S. S. W.) | 50.64 | 49.01 | ----- | .35 | ----- |
| Par-Excellence, (Caulk's) | 46.56 | 49.45 | ----- | 2.06 | 1.93 |
| Arrington's (S. S. W.) | 40.68 | 58.76 | ----- | ----- | .56 |
| Townsend's (S. S. W.) | 41.76 | 57.68 | ----- | ----- | .56 |
| Alba, (S. S. W.) | 41.27 | 58.19 | ----- | ----- | .54 |
| Lawrence, (S. S. W.) | 46.62 | 47.84 | ----- | 4.54 | 1.54 |
| J. & L., (G. & P.) | 46.51 | 52.09 | .36 | ----- | 1.04 |
| Welch's, (G. & P.) | 40.16 | 51.86 | ----- | ----- | 1.47 |
| F. & Y. (Standard) | 44.65 | 49.02 | ----- | 3.82 | 2.52 |
| American | 45.43 | 52.15 | ----- | ----- | 2.22 |
| Vose's Crown | 43.12 | 51.41 | ----- | 2.61 | 2.86 |
| Century | 46.82 | 51.86 | ----- | ----- | 1.32 |
| Success. (R. & R.) | 49.00 | 49.10 | ----- | ----- | 1.90 |
| Pyramid (F. & G.) | 49.36 | 45.23 | ----- | 2.93 | 2.28 |
| Pearl (Dearborn Co.) | 49.77 | 47.05 | .40 | 1.86 | .92 |
| Martin's Gold | 52.37 | 43.71 | .56 | ----- | 2.36 |
| High Stand (F. & G.) | 55.34 | 41.34 | ----- | 1.05 | 2.27 |
| Hammond's Gold | 56.30 | 39.67 | .75 | ----- | 3.28 |
| J. & L. Velox | 55.24 | 38.42 | ----- | 3.81 | 2.53 |
| *Flagg's Contour (S.S.W.Co.) | 55.09 | 42.97 | ----- | ----- | 1.95 |
| †Flagg's Sub-Marine | 52.16 | 44.68 | ----- | 3.16 | ----- |

*Flagg's Contour formula, as stated by Dr. Flagg, should be silver, 63.00, Gold, 3. tin, 34.

†Flagg's Sub-marine, as stated by Dr. Flagg, should be silver, 55. copper, 5. tin 40.

| | | | | | |
|---|---|---|---|---|---|
| F. & G., Euodon | 60.08 | 33.80 | ----- | 3.79 | 2.33 |
| Hewitt's Sterion | 61.89 | 31.85 | ----- | 4.16 | 2.10 |
| Dr. Kester's 5% gold | 67.46 | 26.24 | 5.14 | ----- | 1.16 |
| F. & Y. Permaneuo | 66.59 | 24.94 | ----- | 3.91 | 4.57 |
| True Dent (S .S. W. Co.) | 65.73 | 27.69 | ----- | 4.30 | 2.28 |
| Fellowship | 67.71 | 27.95 | ----- | 3.18 | 1.16 |
| Acme (Garhart's) | 68.00 | 27.00 | ----- | 3 00 | 2.00 |
| 20th Century | 66.09 | 27.38 | ----- | 4.07 | 2.46 |
| Rego (Sibley) | 66.13 | 27.29 | ----- | 4.57 | 2.01 |
| Micrometric (R. & R.) | 67.14 | 26.64 | ----- | 4.31 | 1.91 |
| D. & H. "Contour" | 51.91 | 45.87 | .97 | ----- | 1.25 |
| D. & H. "Sub-Marine" | 48.95 | 45.33 | ----- | 4.24 | 1.48 |
| Page's Perfect | 56.49 | 38.12 | ----- | 1.13 | 4.29 |
| Dawson's (Consolidated Mfg. Co's Formula) | 48.82 | 49.23 | ----- | ----- | 1.95 |
| Garhart's High Standard | 60.00 | 38.00 | ----- | 1.00 | 1.00 |

You will find that human nature is about the same in all walks of life. There are manufacturers that take as much pains in the manufacture of their products as you do in your professional work. On the other hand, we have another class that aspire to nothing save the production of a second-grade article which they represent to be of the very first quality. We also have the manufacturer that produces alloys of good formulæ, but makes them in a careless and slovenly manner. They are lacking in homeogeneity of formulæ and cut as well as those various details of manufacture so necessary for the production of a high-grade alloy. Alloys of first quality do not combine readily with mercury and as a rule set rather rapidly. The makers of inferior goods take advantage of these properties which they endeavor to impart to their own cheap products. In order to imitate the working and setting properties of a high grade alloy, they "pad" their low silver formulæ with large amounts of copper and zinc. I am not in position to state that large percentages of copper and zinc are particularly injurious to alloys, although will say that such alloys are very weak in edge-strength and discolor very quickly in the mouth. Amalgams made from pure silver and tin are insoluble in the secretions of the mouth while those made from copper and zinc are just the reverse. It is not at all improbable that the use of large percentages of copper and zinc in a formulæ containing low percentages of silver, may in the course of time be affected by the saliva. The quick-setting alloys appear to be out of the range of the man who counterfeits. It is possible to produce a quick-setting alloy from a forty-five percent silver formulæ. The rapid-setting features may be obtained as well as the slow-mixing properties. Alloys of this nature are very weak in edge-strength.

It is reasonable to suppose that if an alloy is homeogeneous in formula that it should be homeogeneously combined with mercury. Experience has taught us that the higher grade alloys retard the action of mercury, hence the more reason why they should be intimately mixed before introducing them into the cavity. Thoroughly mix your alloy, having no fear of a faint surplus of mercury being present when first forming the mix. Use gentle pressure in removing the excess mercury after the metals have combined. Return the mass to the palm of the hand and knead vigorously until it has softened, after which operation you may wafer as before. Now repeat the wafering and kneading processes until four operations all told have been completed. If you are using a medium-setting alloy, divide the mass into several portions and wafer each one separately, using the chamois and plier method. Quick-setting alloys should not be wafered so excessively dry, the remaining surplus of mercury should be worked out while inserting the amalgam into the cavity.

If you were afflicted with nose or throat trouble, you would not place your case in the hands of a general practitioner. It would be necessary for you to go to a specialist, one who was qualified in the way of experience, knowledge and office equipment so necessary to look after your case in the most scientific manner. The alloys you use in your practice should bear the stamp of some reliable manufacturing concern, preferably a firm that has made this particular branch of the dental business a specialty. The manufacture of high-grade alloys is now down to a mathematical science so far as the better class of manufacturers are concerned. In order to attain a proper degree of proficiency, not only skill, but a high-grade equipment of especially-designed cutting machines, smelting furnaces, annealing ovens and instruments of precision for standardizing the finished product are needed.

One of our illustrious presidents, Grover Cleveland, said "It is a conditiou and not a theory that confronts us." We have been indulging in too many speculative theories as to why we have so many failures in saving teeth when using amalgam as a filling material. To begin with, we must concede the fact that the tooth structure of the hardy pioneers was by far healthier and less susceptible to decay than the tooth structure of the present-day generation. We are all familar with Darwin's works. He has called our attention to the gradual change which takes place in all forms of animal life and which is so necessary to cope with in the changing conditions of our environment. It will be seen that we have improved methods of dental surgery, appliances and filling materials, all of which are offset by a weakened condition of the tooth structure.

Generally speaking, why do so many amalgam fillings fail? I am referring to the ordinary, every-day class of failures which are a never-ending topic of conversation. It occurs to me that the people are getting what they are paying for, and nothing else. When they grow wiser and find that cheap amalgam work does not pay, we will hear much less about "shrinkage," wide margins, etc. Considering the average price paid for an amalgam filling, we must admit that the proper effort is not put forth in preparing and filling the cavity, such as the dental profession would do if they received a just compensation for their work.

The same care and discretion are required in preparing a cavity as if the filling were of gold. Matrices where indicated are often discarded and the amalgam is not thoroughly mixed nor wafered, It is plastered in the poorly-prepared cavity as you would spread butter on your bread. It is simply another case of "American haste," which seems to be necessary if you are ever going to come out ahead of the great "amalgam game."

The people are not at fault because they want cheap dentistry. Now what does the public know about dentistry? Why don't you take the public into your confidence and teach them the difference between good and poor dentistry? You must reach them at their homes or where they are in the mood to think it over. It would be a very humane procedure, since it would result in saving of more teeth and pain as well as obtaining better fees for yourself. The people have no time for argument when they are in pain, which is a good reason why you can not educate them while they are in your office. You want the best of everything when equipping your office. You are willing to pay the price so long as you are convinced that such an article is better than the rest. The public follow the same lane; they want the best. So long as all amalgam fillings look alike to them, they will always pay the lowest prices, and dentists will buy poor amalgams.

The high-grade alloys of to-day will do their work if you will do yours. You can't take too much time in preparing your cavities nor use too much pressure when placing the properly-prepared amalgam into those cavities. You should use a matrix where indicated and never mix more quick-setting alloy than is necessary to fill one cavity. Never use a quick-setting amalgam after it has begun to harden. Throw it away, and if necessary make two mixes for contour fillings. Polish your fillings to the extent that they will not reveal any scratches when viewed through a hand magnifying glass. I should not use any alloy that carrier less than sixty percent of silver. Don't worry about color providing that you take the pains to polish your fillings. Color chiefly depends upon the hardness of your filling, providing it has been properly polished at least twenty-four hours after the amalgam has been inserted into the cavity. The fillings that hold their color the best are invariably those which have been exposed to the stress of mastication. You will understand that they are polished a least three times a day. Their bright color is not due in any sense to any particular virtue. that is possessed by the alloy, save its high silver formula. The presence of gold and platinum will not prevent shrinkage nor keep your alloy from discoloring. Gold plate under 22k will discolor in the mouth. Good sense ought to tell us that the same percentage of gold is required to preserve the color of amalgams. It will be seen that they have to carry from ninety to ninety-two percent of pure gold, hence there would not be any room left for the other necessary metals.

Do not overlook the fact that you cannot see all of fillings shrinkage with the naked eye. While the margins may look perfect, the microscope will reveal the presence of a fair amount of space. It is for this reason that we use a specially-constructed micrometer for detecting and recording such small spaces as are invisible to the naked eye, when testing in amalgam manufacture.

DISCUSSION.

Dr. M. L. Ward: Mr. Garhart certainly has rendered the profession a great service in going to the expense and trouble of analyzing so many of the amalgams offered as reliable preparations for filling teeth. Whether his analyses are correct or not he has at least given his own formulas, and I believe from analyses I myself have made that these he gives us to-day are substantially correct. There is no good reason why manufacturers should not give out their formulas. The secret of a good amalgam is not dependent altogether on its formula, but upon the method of manufacture. I do not mean to say that a good and careful manufacturer could make an acceptable amalgam from a poor formula. But I would say emphatically that a careless manufacturer can not hope to make a good amalgam with the best formula. And I would suggest that the only thing the profession can do is to buy such amalgams as have back of them reliable and intelligent producers. The great mistake the profession is making is that every itinerant salesman that comes along with amalgam that he offers at a few cents less than reliable makers ask is encouraged by his ability to sell his goods. "All coons are alike" to too many practitioners.

There are a good many amalgam secrets yet unknown, not manufacturers' secrets, but chemical and physical secrets. Dr. Black has done a great work and others have added their evidence, but we have just begun to open up this subject and the profession must do the delving, as no one else is so much interested. There is good evidence at hand to indicate that amalgams are not true chemical compounds. What then are they? How does mercury serve to bind the particles of alloy together? Some claim it acts largely as a cement and evaporating allows the hardened mass to change form months after it has set. This is a question we should find an answer for. Some alloys seem to work well when freshly cut, but after aging they prove very bad as stoppings. I recently tested eleven of the cheaper alloys purchased on the market and found that nine shrank badly and two expanded considerably. Alloys that can not be aged without deterioration in quality are certainly made from badly-constructed formula. Such alloys if not used very soon after made will not give good results. All alloys should be artificially aged and tested for changes before they are put on the market. Few manufacturers take the trouble to do this, and the result is a poor amalgam, or at least one that is unreliable in results. If we could decide what was the best formula for an alloy and then how it should be manufactured to get the best results, we could then trust our reliable manufacturers to make for us a product that would give good results. I have no doubt as Mr. Garhart has said much of amalgam work fails because the dentist does not do his part faithfully. There is perhaps as much censure to be placed on his shoulders as on the manufacturers. We must re-study amalgam cavity preparation, and methods of packing it to make any advance.

Dr. Hines: I have used as many alloys as four or five during the part five years, and can't say that I have noticed much difference as to results. I make it a point to prepare my cavity with great care, and use the dam and matrix where indicated. I pack alloys carefully, using small pieces and forcing each one into place and working out all the mercury by using as much force as possible to avoid air spaces or bridging. I also have my patients return when the amalgam has set, and I then thoroughly polish the surfaces.

Dr. Ward was asked to name some of the more reliable amalgams on sale. This he declined to do; but said that those containing at least sixty-five percent of silver and which were carefully made were liable to give the best results. He did not think either gold or platinum add materially to the value of amalgams.

